# Pressure dependency of localization degree in heavy fermion CeIn_3_: A density functional theory analysis

**DOI:** 10.1038/srep31734

**Published:** 2016-08-24

**Authors:** M. Yazdani-Kachoei, S. Jalali-Asadabadi, Iftikhar Ahmad, Kourosh Zarringhalam

**Affiliations:** 1 Department of Physics, Faculty of Sciences, University of Isfahan (UI), Hezar Gerib Avenue, Isfahan 81746-73441, Iran; 2 Center for Computational Materials Science, University of Malakand, Chakdara, Pakistan; 3 Abbottabad University of Science and Technology, Havelian, Pakistan; 4 Department of Mathematics, University of Massachusetts Boston, Boston, MA 02125, USA

## Abstract

Two dramatic discrepancies between previous reliable experimental and *ab initio* DFT results are identified to occur at two different pressures in CeIn_3_, as discussed through the paper. We physically discuss sources of the phenomena and indicate how to select an appropriate functional for a given pressure. We show that these discrepancies are due to the inaccuracy of the DFT + U scheme with arbitrary U_eff_ and that hybrid functionals can provide better agreement with experimental data at zero pressure. The hybrid B3PW91 approach provides much better agreement with experimental data than the GGA + U. The DFT + U scheme proves to be rather unreliable since it yields completely unpredictable oscillations for the bulk modulus with increasing values of U_eff_. Our B3PW91 results show that the best lattice parameter (bulk modulus) is obtained using a larger value of α parameter, 0.4 (0.3 or 0.2), than that of usually considered for the AFM phase. We find that for hybrid functionals, the amount of non-local exchange must first be calibrated before conclusions are drawn. Therefore, we first systematically optimize the α parameter and using it investigate the magnetic and electronic properties of the system. We present a theoretical interpretation of the experimental results and reproduce them satisfactorily.

Cerium based materials have recently received considerable attention due to their interesting properties^1^,^2^,^3^,^4^. It is believed that magnetic interactions can play crucial roles in appearance of these properties^5^,^6^,^7^,^8^. Density functional theory (DFT) have been extensively used as an appropriate approach to investigate the magnetic properties and electronic structures of these materials^9^. However, the predictions of this *ab initio* theory sometimes are so substantially inconsistent with experimental data that cannot be only attributed to the accuracy of the performed DFT calculations. For instance, here we identify two dramatic discrepancies between previous reliable experimental and accurate theoretical DFT + GGA results for CeIn_3_ at two different pressure regimes. The first discrepancy is concerned with the Néel temperature, T_N_, of the compound under question. The previous experimental results showed that the T_N_ of the compound was pressure dependent and completely suppressed at critical pressure P_c_ = 2.5 GPa^10^. However, the previous theoretical DFT + PBE-GGA calculations yield P_c_ ≈ 16 GPa, which is very far from the experimental value^11^. Our DFT + PBE-GGA calculations corroborate this inconsistency between these experimental and theoretical critical pressures. This inconsistency was improved^11^ by WC-GGA functional to a better value of 9 GPa. However, this pressure is also still much larger than the experimental value. The second discrepancy is concerned with the total magnetic moment (TOT) of the compound. The previous DFT + GGA also drastically failed to reproduce the TOT of the system at zero pressure. The PBE-GGA calculations^11^,^12^ predicted the TOT of this compound to be ~0.16 *μ*_*B*_ per Ce atom while the experimental value^13^ was 0.65 *μ*_B_. In contrast to the critical pressure, WC-GGA not only could not improve the TOT, but also made it worse^11^ compared to the experiment and reduced it to 0.11 *μ*_B_. In this paper, we show that the theoretical results are drastically far from the experimental results due to the high pressure dependency of the Ce 4f-electrons localization. We attribute these discrepancies to high sensitivity of the degree of Ce 4f-electrons localization in the compound under study to an exerted external pressure.

To make progress in understanding the basic physics behind these discrepancies more calculations with a variety of functionals are required. A. J. Ochoa-Calle and coworkers by performing systematic DFT calculations on the *ε* and 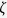 high-pressure solid phases of oxygen employing several exchange-correlation functionals very recently showed that the rather strong dynamical correlation effects and the non-negligible exchange effects had to be taken into account along with electronic delocalization to provide an accurate account of the evolution of the structural properties versus pressure^14^,^15^,^16^. Therefore, in the present work, we also apply the LDA, GGA, LDA + U^17^ and hybrid^18^,^19^ functionals to investigate the magnetic and electronic properties of CeIn_3_ in terms of pressure. Our calculations show that the LDA functional due to its low degree of localization can remarkably improve the first discrepancy by providing the suppressed pressure of T_N_ in close agreement with experiment. However, LDA functional not only cannot improve the second discrepancy but also more deteriorates it by predicting worse TOT at zero pressure than that of GGA. On the contrary, the more advanced band correlated methods (e.g, LDA + U and hybrid functionals) due to their high degree of localization improve the second discrepancy by describing the TOT satisfactorily at zero pressure in agreement with experiment. However, this time the strongly correlated LDA + U and hybrid functionals not only fail to improve the first discrepancy but also make the suppressed pressure worse compared to the previously reported GGA results. These evidences reveal that similar to previously reported GGA results, the other functionals also fail to cure both of the discrepancies at the same time. In this paper, we physically discuss what happens to the system by imposing pressure and how pressure can change the nature of the Ce 4f localization as well as why none of the functionals alone can successfully treat both of the discrepancies simultaneously. This study shows that the Ce 4f-electrons behave differently in different pressures and hence cause the discrepancies to occur.

## Computational Details

All the calculations presented here are performed in the framework of the density functional theory^20^,^21^ by employing the full potential linearized augmented plane wave (LAPW) method^22^,^23^, as embodied in the WIEN2k code^24^, in the presence of the spin-orbit coupling (SOC). Since we intend to investigate the compound under question in its AFM phase, we construct a 2 × 2 × 2 supercell which contains two nonequivalent Ce atoms, one with spin up (Ce^↑^) and the other with spin down (Ce^↓^). We consider a mesh of 182 special *k* points in the irreducible wedge of the first Brillouin zone that corresponds to the grids of 12 × 12 × 12 in the Monkhorst-Pack scheme^25^ for the supercell. We choose the muffin-tin radii (R_MT_’s) to be 2.2 a.u. for In and 2.8 a.u. for Ce. The cutoff parameters K_max_ = 7/R_MT_ and *l*_max_ = 10 are used for the expansions of the wave functions inside the muffin-tin spheres and the interstitial region in terms of the lattice harmonics and the plane waves, respectively. The periodic charge density and potential are Fourier expanded up to G_max_ = 16 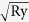. In addition to the LDA and GGA approximations, we use the LDA + U approach with several U_eff_ and B3PW91 hybrid with several *α* as well as B3LYP functionals. In the hybrid functionals generally some portion of the exchange term in the semilocal functional is replaced by the Hartree-Fock (HF) exchange so that general form of these functionals can be written as:


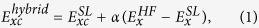


where *α* is a parameter which determines the portion of the Hartree-Fock exchange, 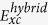 and 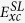 are the hybrid and semilocal exchange-correlation functionals, as well as 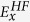 and 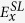 are Hartree-Fock and semilocal exchange functionals, respectively. The B3PW91 and B3LYP hybrid functionals (which we have used in this work) are:


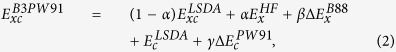



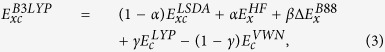


where 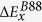 is the Becke’s 1988 gradient correction to the exchange functional^26^, 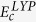 is the correlation functional of Lee, Yang and Parr^27^, 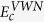 is the VWN local-density approximation to the correlation functional^28^, and 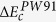 is the Perdew-Wang gradient correction for the correlation functional^29^. Becke optimized the coefficients to *α* = 0.2, *β* = 0.72 and *γ* = 0.81^18^. In this work we allow to vary the *α* parameter in the range of 0 to 0.4 to seek better optimization for our case. One would notice that the LDA + U and hybrid can be considered as semiempirical schemes since by varying the adjustable U parameter in LDA + U and *α* parameter in hybrid schemes one can try to tune the methods to predict more closely some experimental data while deviating more for other data. Thus, in this case it is important to optimize these parameters by taking different physical quantities into account and making sure that the optimized parameters can optimally reproduce the experimental data corresponding to the different physical quantities. In structural properties section, we will follow this strategy and optimize the *α* parameter in B3PW91 to obtain both lattice parameter and bulk modulus in closer agreement with the experimental data. In magnetic properties section, we will show that our optimized *α* parameter can predict also the magnetic moment in better agreement with the experiment compared to the other values of the *α* parameter.

### Structural properties

**Figure 1:**
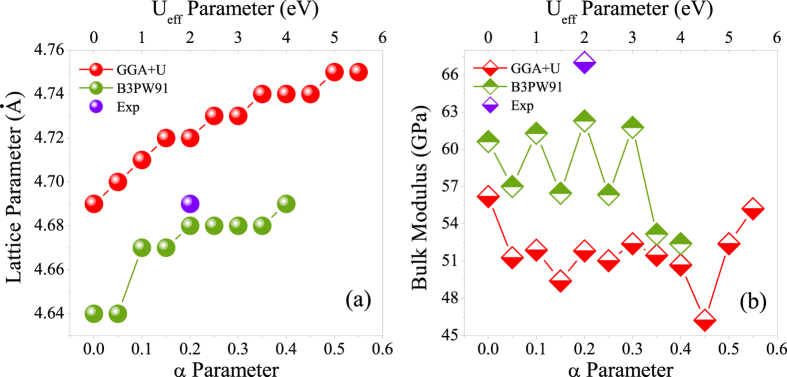
(a) Lattice parameter and (b) bulk modulus of CeIn_3_ in the antiferromagnetic phase as functions of U_eff_ parameter in GGA + U functional and *α* parameter in B3PW91 hybrid functional together with experimental data for comparison.

Here, we intend to evaluate the accuracy of the B3PW91 hybrid functional^18^,^19^ as our selected successful method in the rest of this work in predicting the lattice constant and bulk modulus of the CeIn_3_ compound in the FM and AFM phases. To this end, the results, as tabulated in Table 1, are compared with the experimental data and theoretical results reported by the others. However, one should keep in mind that the correctness of a theoretical description can be only partially validated by comparison with the experimental data or, eventually, by comparison to theoretical results obtained with significantly more sophisticated methods. The structural properties are obtained by calculating the total energies of the AFM primitive unit cell^30^ (see computational details section) as a function of its volume and fitting the data with the Birch-Murnaghan isothermal equation of state^31^ within a variety of exchange-correlation functionals. The results together with the previous experimental^32^,^33^ and theoretical^11^ data are presented for comparison in Table 1. The calculated lattice constants show that their predicted values are not very sensitive to the magnetic ordering as imposed in FM and AFM phases, see Table 1. Hence, we may only discuss the AFM results. The results show that the predicted lattice parameter with the PBE-GGA is larger than those of LDA and WC-GGA. Although our calculated lattice parameter by PBE-GGA is surprisingly identical to the experimental value, PBE-GGA is not successful enough in prediction of bulk modulus compared to that of WC-GGA. In order to simultaneously reproduce the experimental bulk modulus as well as lattice parameter, we use the so called LDA + U and hybrid approaches as the other alternatives. In these methods the degree of localization can be tuned by their adjustable parameters, i.e., U_eff_ in LDA + U and *α* in hybrid schemes. M. Caffarel and coworkers recently showed that the spatial distribution of the DFT spin density distribution was very sensitive to and depended on the fraction of HF exchange used in the functional employed for CuCl_2_ molecule having a single unpaired electron^34^. They showed that for providing a meaningful description for this physical quantity in this molecule using the B3LYP-DFT, the percentage of HF exchange used had to be increased up to about 40%. Therefore, we perform a systematic exploration of the role of the arbitrary value of U_eff_ in GGA + U and dependence of *α* in B3PW91 functional schemes for the AFM phase of the system. In order to provide a better understanding of the results, lattice parameter and bulk modulus are also presented in Fig. 1(a,b) showing how these basic properties change when U_eff_ in GGA + U and *α* in B3PW91 vary. The lattice constant is more increased by increasing the U_eff_ of the GGA + U in worse agreement with the experimental data than PBE-GGA, see Table 1 and Fig. 1(a). The results also reveal that the bulk moduli predicted by GGA + U with all of the values considered for its U_eff_ parameter are farther away from the experimental result compared to that of PBE-GGA, see Table 1 and Fig. 1(b). This indicates that the effects of GGA + U on the lattice constant and bulk modulus are in opposite directions of the results improvement over the PBE-GGA results. If we change the base of the exchange-correlation energy from PBE-GGA to LDA and use LDA + U instead of GGA + U, however, we can anticipate to overcome the problem. Our results verify this anticipation, because as can be seen from Table 1 the directions of LDA + U improvement are opposite to that of GGA + U and thereby corrected towards better predictions of LDA + U than LDA. The reason is that the LDA lattice parameter (bulk modulus) is lower (higher) than that of the experimental value and using LDA + U causes to increase (decrease) the LDA lattice parameter (bulk modulus). Dependence of the lattice parameter to the correlations among 4f-electrons is in agreement with the previous results for the isostructural *α*- and *γ*-Ce compounds. The GGA + U with U_eff_ = 4.4 eV (PBE-GGA) predicts^35^ the larger (smaller) lattice parameter of the fcc *γ*-Ce (*α*-Ce) in agreement with experiment. The B3LYP with *α* = 0.2 lattice parameter is larger and bulk modulus is smaller than those of GGA + U. Therefore, the onsite hybrid B3LYP functional only aggravates the unsatisfactory GGA + U results and thereby yields the worst presented results in Table 1. In the hybrid B3LYP functional, LYP and VWN5 correlation functionals are used, while in the hybrid B3PW91 functional PW91-LDA and PW92-LDA correlation functionals are used. As discussed above, the overall effect of the included correlations in LDA + U is more successful than that of GGA + U for the system under study. From this we can anticipate that the overall effect of the included correlations in B3PW91 due to its included PW91-LDA and PW92-LDA terms can be also more efficient than that of B3LYP functional for the compound under study. This anticipation is confirmed by the results presented in Table 1 and Fig. 1. According to results presented in Table 1 and Fig. 1(a), the lattice parameter increases as *α* parameter increases with the B3PW91 XCF. However, this is not the case for the bulk modulus, see Fig. 1(b). The bulk modulus of the system, as shown in Fig. 1(b), fluctuates as *α* parameter increases from zero to 0.4 by step 0.05. The B3PW91 XCF with *α* = 0.4 predicts the lattice parameter in the best agreement with the experimental result compared to other values of *α*, however, it fails to satisfactorily predict the bulk modulus. Figure 1 shows that the best agreement between theory and experiment may not be necessarily achieved only by increasing the *α* parameter and both of the lattice parameter and bulk modulus would be taken into account for optimizing the *α* parameter. We also calculate the structural properties using the LDA-EECE method. However, this method, as shown in Table 1, is not very successful in prediction of CeIn_3_ lattice constant and bulk modulus. The phase transition pressure and coexistence pressure range were predicted^14^,^16^ for *ε* and 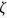 solid phases of O_2_ by B3PW91 with 20% of HF exchange to be 75 GPa and 75–145 GPa in an acceptable agreement with the experimental data^36^ of 95 GPa and 95–110 GPa, respectively. Structural parameters of the *ε* and 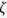 phases were also predicted^14^ by B3PW91 in excellent accordance with experiments. These reports in agreement with our results show that the performance of hybrid functional methods strongly depends on the amount of non-local HF exchange. Now, by taking the above discussed points into consideration, the time seems apt to conclude this section by attaining without proof that the overall effects of the hybrid B3PW91 functional with *α* = 0.2 on the structural properties of the CeIn_3_ compound are more impressive and better than the other functionals.

### Magnetic properties

**Figure 2:**
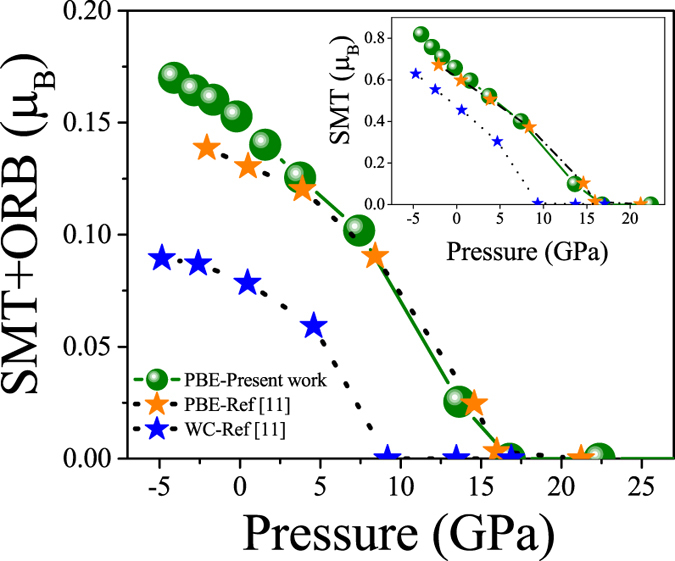
Ce spin magnetic moment inside muffin-tin sphere plus orbital magnetic moment (SMT + ORB) calculated by PBE-GGA functional at several pressures accompanied by other theoretical results. Inset shows the SMT contributions.

It is generally believed that magnetic ordering can be changed by pressure^10^,^37^,^38^,^39^. This is the case for the compound under question too, since experimental results^10^ show that the magnetic ordering Néel temperature, T_N_, of the CeIn_3_ compound decreases by applying pressure. The previous theoretical calculations^11^ using PBE-GGA functional also confirm this fact that the magnetic moments of CeIn_3_ decrease by imposing pressure. Our PBE-GGA calculations also validate the latter theoretical results, as shown in Table 2 and Fig. 2. So far everything has been qualitatively fine as expected. But, two sizable discrepancies can be observed when the theoretical predictions are quantitatively compared with the experimental data. The first discrepancy is concerned with the T_N_ of the compound. The experimental results show that the T_N_ of CeIn_3_ compound decreases with increasing pressure and may be completely suppressed at the critical pressure P_c_ = 2.5 GPa, viz. 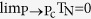
10. However, previous and our PBE-GGA results show that the TOT decreases as pressure increases and eventually vanishs at suppressed pressure P_s_ = 16 GPa, viz. 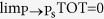, (see Table 2 and Fig. 2). A comparison elucidates that the theoretically predicted P_s_ = 16 GPa by PBE-GGA is much larger than the corresponding experimental P_c_ = 2.5 GPa. It was tried to improve this discrepancy by using the WC-GGA functional^11^. Although the theoretical WC-GGA P_s_ = 9 GPa is closer to the corresponding experimental P_c_ = 2.5 GPa, the predicted P_s_ using the WC-GGA functionals is still very far from P_c_. The second discrepancy is concerned with the TOT of the system. Reliable neutron-diffraction results show that the TOT of the CeIn_3_ is 0.65 ± 0.1 *μ*_*B*_ at T = 5 K and zero pressure^13^. However, previous theoretical study^11^ at zero pressure showed that the TOT was 0.170 *μ*_*B*_ within PBE-GGA and 0.112 *μ*_*B*_ within the WC-GGA. Our PBE-GGA calculations lead to the TOT = 0.24  *μ*_*B*_ at zero pressure which is fairly comparable with the previous PBE-GGA result. In addition to spin magnetic moment per atom in the muffin-tin shpere, we have also considered and included the spin magnetic moment per atom in the interstitial region and this is why our calculations a little bit differ from the previous theoretical work. Regardless of the latter small difference between theoretical results, a comparison elucidates that the theoretically predicted TOT’s by PBE-GGA and WC-GGA are much smaller than the corresponding experimental TOT = 0.65 *μ*_*B*_. Therefore, these functionals are not enough successful in predicting the TOT at zero pressure.

As discussed above there are two dramatic inconsistencies between theory and experiment: 1) theoretical P_s_ is far from the experimental P_c_, and 2) there are a large discrepancy between the theoretical and experimental TOT’s at zero pressure. We show that the discrepancies between experiment and theory originate from high pressure dependency of the Ce 4f-electrons localization. On the other hand, the validity of the selected exchange-correlation functional for simulating CeIn_3_ are highly sensitive to the pressure regime at which the DFT calculations are performed. Therefore, these discrepancies cannot be well resolved simultaneously only by increasing the accuracy of the calculations within a single specific currently available functional as they occur in two different pressure regimes.

The main idea to overcome these inconsistencies is inspired from the following experimental observations. The dHvA experiments revealed that the character of 4f-electrons was changed from localized to itinerant above the P_*c*_^40^,^41^. These results elucidate that the degree of localization can play important roles in the properties of this compound near the P_*c*_. This has motivated us to investigate the effects of the localization degree of Ce 4f-electrons and pressure on the magnetic properties of CeIn_3_. For this purpose, we calculate magnetic moments per Ce atom of this compound versus pressure within several exchange-correlation functionals (XCFs). These XCFs contain PBE-GGA + U with varying U_eff_ from 0.5 to 5.5 eV by step 0.5 eV, B3PW91 hybrid functional with varying *α* from 0 to 0.4 by step 0.05, B3LYP hybrid functional with *α* = 0.2, LDA, LDA-EECE, and PBE-GGA. The degree of localization is adjusted by U_eff_ and *α* parameters of the GGA + U and hybrid methods, respectively. Our results are presented in Table 2 together with experimental^13^ and other theoretical^11^,^12^ results for comparison.

The GGA + U results presented in Table 2 show that TOT decreases as pressure increases. The same result can be seen for B3PW91 and B3LYP. Even though it is possible (at least in principle) to find the P_s_ using the GGA + U, B3LYP and B3PW91, we anticipate that the P_s_ using these functionals must be much larger than experimental P_c_. This anticipation can be verified by the results given in Table 2, since TOT is not zero at pressures very higher than P_c_ for all of the considered U_eff_ values using the GGA + U and all of the *α* parameters with the B3PW91. Consequently, like GGA + U, the hybrid functionals cannot also predict the P_s_ in acceptable agreement with experiment. These results indicate that increasing the 4f-correlation using GGA + U and hybrid functionals even make worse the predicted P_s_ than that of the PBE-GGA with respect to the P_c_. Therefore, the 4f-correlation must be decreased to improve the P_s_ compared to the experimental P_c_. As another alternative, we then use the LDA functional which has the least correlation. The LDA calculations predict that P_s_ = 0, because TOT is zero above the zero pressure, see Table 2. The correlation of LDA is smaller than that of GGA and this enables LDA to substantially improve the P_s_ prediction in much better agreement with the experimental value P_c_ compared to those of PBE-GGA and even WC-GGA as well as the other considered functionals. Hence, LDA could solve the first inconsistency problem between theoretical P_s_ and experimental P_c_ by decreasing the degree of 4f localization.

We now concentrate on the second problem, i.e., the disagreement between theoretical and experimental TOT at zero pressure. The LDA functional leads to zero TOT at zero pressure. Thus, LDA deteriorates the TOT predictions of PBE-GGA and WC-GGA at zero pressure compared to the experiment^13^, while conversely GGA + U and hybrid approaches improve them, see Table 2. The results, as tabulated in this table, show that the B3PW91 with *α* = 0.2 is the most successful functional for the TOT prediction at zero pressure among the other considered functionals, because in this case TOT becomes 0.67 *μ*_*B*_, which is in more agreement with the experimental result^12^ of 0.65±0.1 *μ*_*B*_. As can be seen from the results presented in Table 2, the TOT at zero pressure is not improved by changing the *α* parameter to values more or less than 0.2. Therefore, *α* = 0.2 would be considered as an optimized parameter for B3PW91 in this case in agreement with the discussion presented in structural properties section. Thus, B3PW91 with *α* = 0.2 can cause to overcome the second inconsistency by increasing the degree of localization.

As discussed above, the LDA functional can only predict the P_s_ in good agreement with P_c_, but it is unsuccessful in reproducing the TOT at zero pressure. Conversely, the B3PW91 with *α* = 0.2 can only predict the TOT at zero pressure, but it fails to predict the P_s_ compared to the experimental P_c_. All these indicate that increasing the degree of localization increases the TOT and makes the theoretical prediction consistent with the experimental result at zero pressure. This is in agreement with the recent theoretical work^42^. Conversely, reducing the degree of 4f-localization decreases the predicted P_s_ and thereby decreases the inconsistency between the theory and experiment. Hence, the degree of 4f-localization can influence the effects of pressure on the magnetic properties. Therefore, it is not possible to describe magnetic properties of CeIn_3_ for every pressure only by one fixed degree of 4f-localization. Thus, the degree of localization depends on the pressure and should be tuned in terms of the pressure. High pressure is equivalent to the low 4f localization, and vice versa. This is in agreement with the mentioned dHvA observations which show the changes in the degree of 4f-localization above the P_c_^40^,^41^. These changes have been also observed in the CeSn_x_In_3−x_ using the Gd^3+^ electron resonance experiments at the critical x_c _≈ 0.65 where T_N_ → 0^43^. Since atomic radius of Sn is bigger than the atomic radius of In, increasing the Sn concentration is equivalent to increasing the pressure, which is known as the chemical pressure. Furthermore, the dependency of XCF to the pressure regimes in agreement with our results was also previously observed in *ε* and 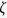 phases of solid O_2_^14^,^16^.

### Electronic structure

**Figure 3:**
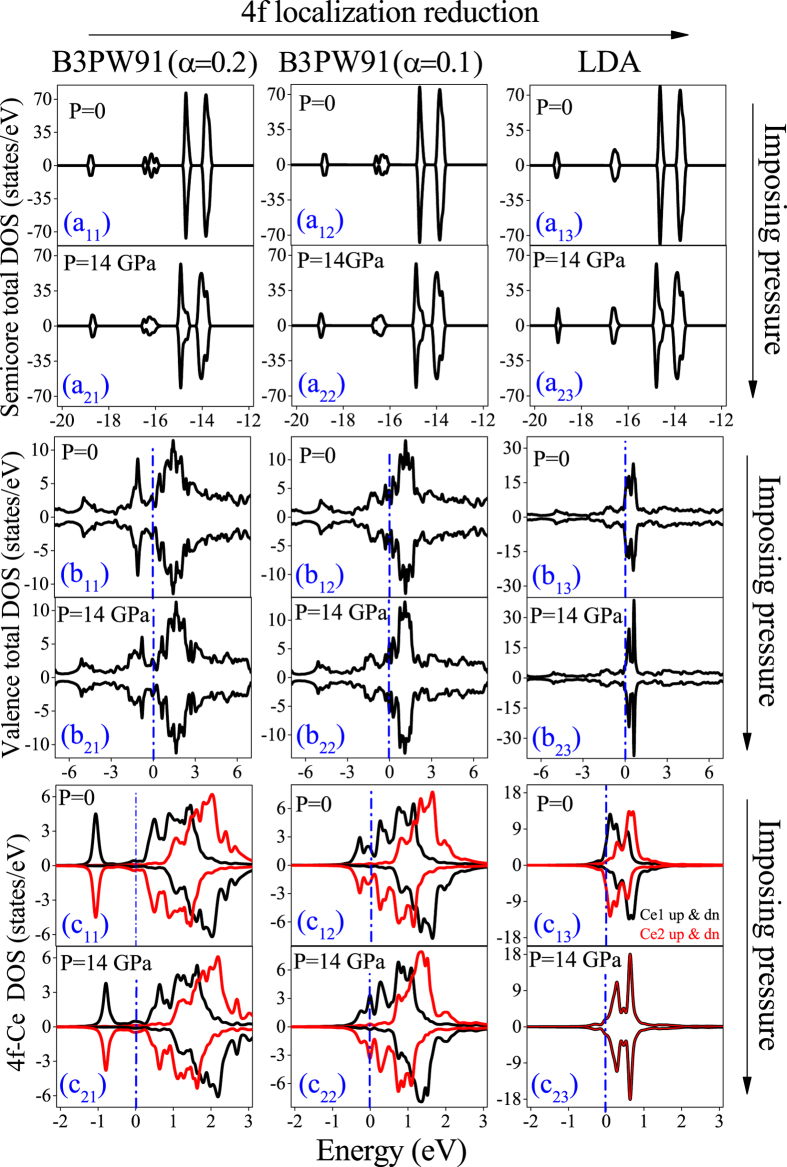
Calculated semicore and valence total DOSs as well as 4f Ce DOSs of CeIn_3_ at P = 0 and P = 14 GPa using the B3PW91 with *α* = 0.2 (a_*i*1_ to c_*i*1_), B3PW91 with *α* = 0.1 (a_*i*2_ to c_*i*2_) and LDA functionals (a_*i*3_ to c_*i*3_), where *i* = 1 and 2. The vertical dashed lines indicate the Fermi level.

**Figure 4:**
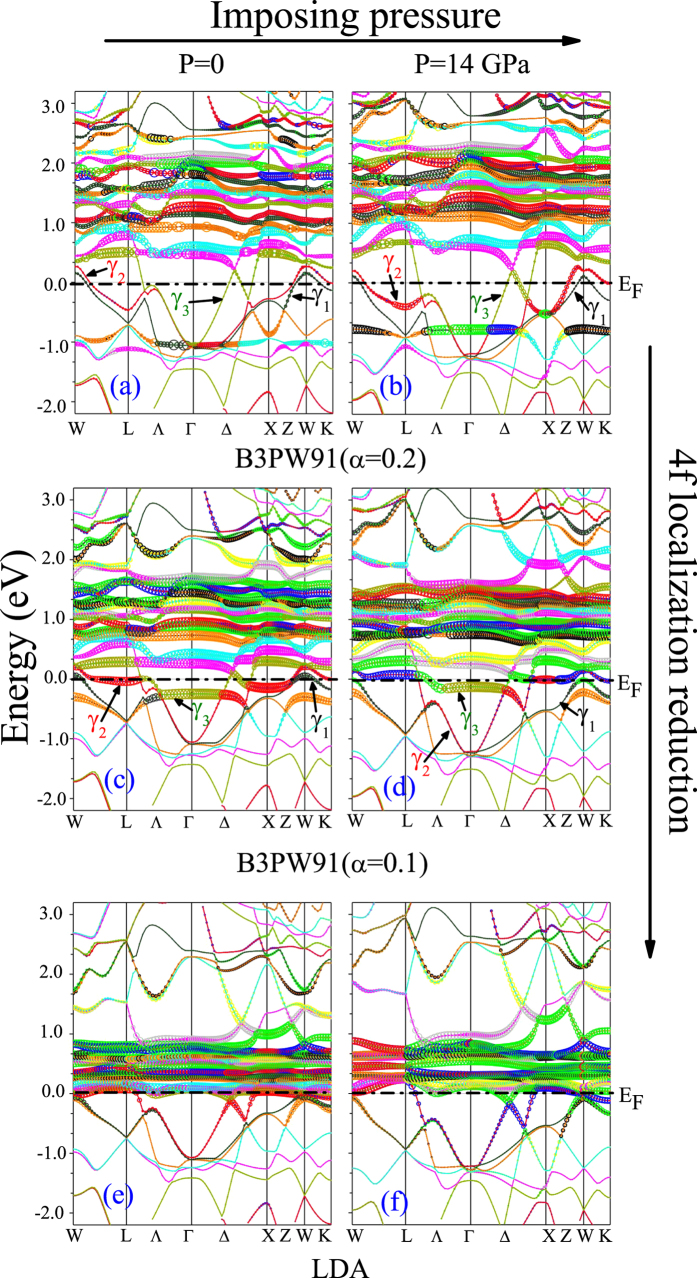
Calculated band structures of CeIn_3_ using the B3PW91 with *α* = 0.2, B3PW91 with *α* = 0.1 and LDA functionals at P = 0 and P = 14 GPa pressures for spin up. The 4f states are also characterized by bands thickness. Larger thickness indicates the more contribution of 4f states.

**Figure 5:**
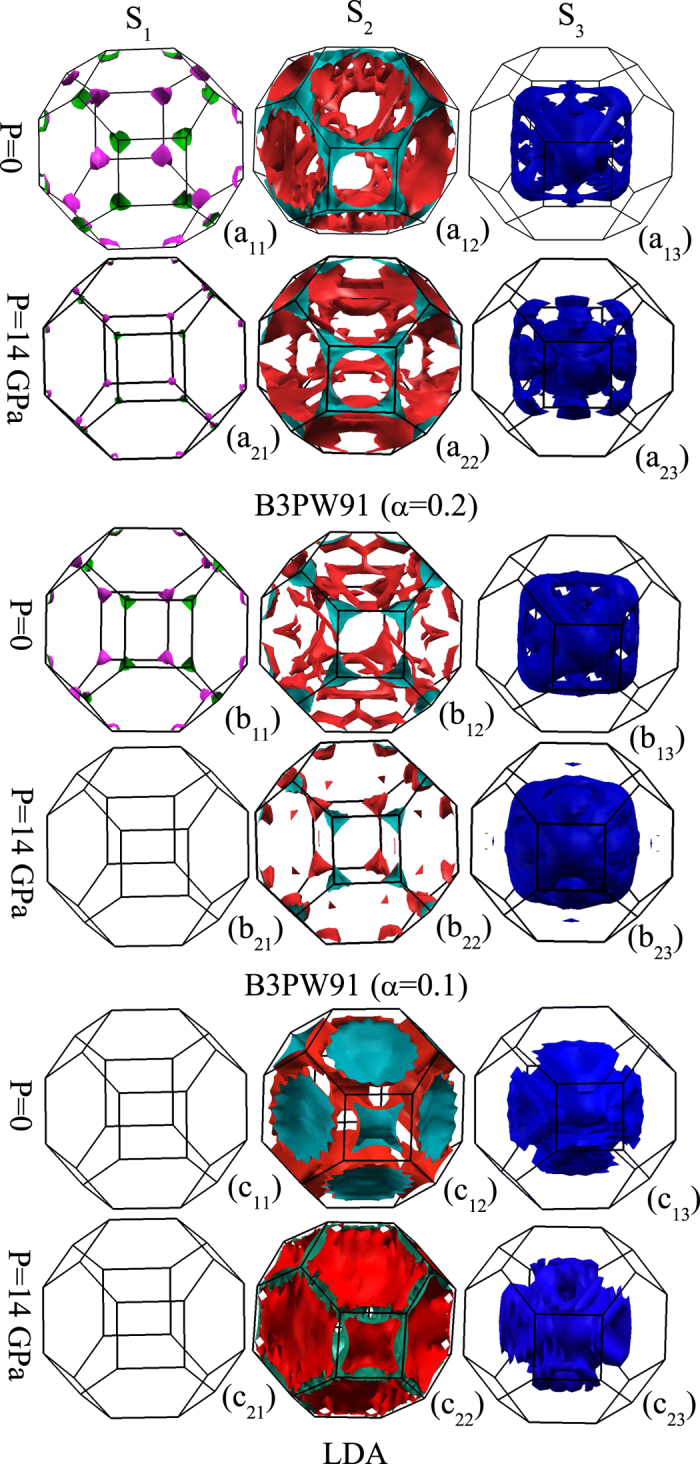
Calculated Fermi surface branches S_1_ and S_2_ as well as S_3_ corresponding to the *γ*_1_ and *γ*_2_ as well as *γ*_3_ bands (seeFig. 4) for CeIn_3_ using the B3PW91 with *α* = 0.2 (a_*ij*_), B3PW91 with *α* = 0.1 (*b*_*ij*_) and LDA functionals (*c*_*ij*_) at P = 0 and P = 14 GPa pressures for spin up, where *i* = 1 and 2 and *j* = 1 to 3; *i*-index refers to the 2 pressures P = 0 and 14 GPa, while *j*-index refers to the 3 branches S_1_, S_2_, and S_3_, all respectively.

As discussed in magnetic properties section, the pressure and degree of 4f-localization can affect the magnetic properties. The pressure effects in turn depend on the degree of 4f-localization. In order to elucidate the effects of pressure and 4f-localization as well as their dependencies on each other, here we turn our attention to the electronic structure of CeIn_3_. To this end, we investigate the electronic structure of CeIn_3_ at two different pressures within three different functionals corresponding to three different degrees of 4f-localization. The selected two pressures are P = 0 corresponding to *a* = 4.68 Å and P = 14 GPa corresponding to *a* = 4.44 Å. These pressures are calculated by B3PW91 with *α* = 0.2. The latter 14 GPa pressure is equivalent to the predicted LDA pressure 3.5 GPa, which is close to the experimental P_c_ = 2.5 GPa. Since we intend to investigate the effects of 4f-localization degree on the electronic properties, three functionals are selected. These functionals are B3PW91 with *α* = 0.2 having high degree of 4f localization, B3PW91 with *α* = 0.1 having intermediate degree of 4f localization, and LDA having low degree of 4f localization. The total and 4f partial DOSs (Fig. 3), band structures (Fig. 4) and Fermi surfaces (Fig. 5) of CeIn_3_ are calculated using these mentioned three methods and two pressures.

### Total and 4f partial DOSs

Total DOSs are shown in semicore [see Fig. 3 (a_ij_)] and valence [see Fig. 3 (b_*ij*_)] regions separately and 4f partial DOSs are also shown in Fig. 3 (c_*ij*_), where *i* = 1, 2 and *j* = 1 to 3. The DOSs calculated by B3PW91 with *α* = 0.2 at zero pressure, as shown in Fig. 3(a_11_), (b_11_), and (c_11_), are in good agreement with those of previously calculated^30^ by GGA + U with U_eff_ = 5.5 eV. There are two peaks in the semicore region near −15 eV and −14 eV energies for the considered three functionals and two pressures, see Fig. 3 (a_*ij*_) (*i* = 1, 2 & *j* = 1 to 3). Pressure causes these peaks to be broadened and decreased in magnitude, as can be seen by comparing Fig. 3(a_11_) with (a_21_), and Fig. 3(a_12_) with (a_22_), as well as Fig. 3(a_13_) with (a_23_). However, pressure has no remarkable effect on the locations of these peaks. The results obtained from the considered three functionals show that the semicore states look precisely alike at P = 0, see Fig. 3 (a_1*j*_) (*j* = 1 to 3). This observation at zero pressure remains correct at P = 14 GPa as well, see Fig. 3 (a_2*j*_) (*j* = 1 to 3). These show that the reduction of the 4f localization cannot significantly affect the semicore states. All these show that the effects of pressure on the semicore states are more considerable than those of the 4f-localization degree. The semicore states are mostly composed of the d-In states while the valence states are mostly made up of 4f-Ce states. Thus, it is expected that the effects of the 4f localization are more considerable on the valence states than the semicore states. The reduction of the 4f localization at zero pressure causes the total DOSs in the valence region to be narrower and pile up with taller height around the Fermi level, see Fig. 3 (b_1*j*_) (*j* = 1 to 3). The same result can be clearly seen at P = 14 GPa by comparing the narrow and piled up DOSs around the Fermi level in Fig. 3(b_23_) with the broad total DOSs shown in Fig. 3(b_21_,b_22_). Although the effects of pressure on the valence states is almost negligible using the B3PW91 with *α* = 0.2 [compare Fig. 3(b_11_) with (b_21_)], the valence DOSs can be also changed by pressure. For example, the LDA results show that the total valence LDA DOS is substantially affected by the pressure, compare Fig. 3(b_13_) with (b_23_). Thus, the effects of pressure on the valence states increase by reduction of the 4f localization. This implies that the effects of pressure on the valence DOS depend on the degree of 4f localization. This dependency is what we have emphasized on it in the magnetic properties of CeIn_3_, see magnetic properties section. The 4f partial DOSs more clearly reveal the pressure dependence of the valence states on the degree of 4f localization. There are two nonequivalent Ce atoms in the considered unit cell. These Ce atoms are labeled as Ce1 and Ce2. The results show that up and down 4f DOSs of Ce1 are asymmetric with respect to each other at zero pressure using all considered functionals, see Fig. 3 (c_1*j*_) (*j* = 1 to 3). This is the case for Ce2 at zero pressure as well, see Fig. 3 (c_1*j*_) (*j* = 1 to 3). These imply that the up 4f DOS of each Ce atom cannot be canceled out by its own down 4f DOS and thereby yields nonzero total magnetic moment per Ce atom. But, the up 4f DOS of the Ce1 cancels the down 4f DOS of the Ce2 out and vice versa which yields zero for the total magnetic moment per unit cell as expected for the AFM phases. The same results are also observed at P = 14 GPa using the B3PW91 with *α* = 0.2 and 0.1, see Fig. 3 (c_2*j*_) (*j* = 1 to 3). However, an interesting different behavior is observed at P = 14 GPa using LDA, see Fig. 3(c_23_). In contrast to the LDA 4f DOSs at zero pressure, the LDA 4f DOSs at P = 14 GPa show that up and down 4f DOSs of Ce1 are symmetric with respect to each other. Thus, the up 4f DOS of each Ce atom can be canceled out by its own down 4f DOS and thereby yields zero total magnetic moment per Ce atom. Therefore, LDA predicts that CeIn_3_ would behave as a paramagnetic (PM) system at the considered pressure (14 GPa). But, one should not here conclude that the phase transition form AFM to PM occurs at 14 GPa, since LDA results in Table 2 clearly show that the total magnetic moment per Ce atom and its components are all zero for every pressure P ≥ 0. Thus, the LDA predicts that the AFM to PM phase transition occurs at zero pressure. All these indicate that both the pressure and the degree of 4f localization must be simultaneously taken into consideration for a theoretical investigation of the AFM to PM phase transition in CeIn_3_ which evidently exhibits the dependence of pressure effects on the 4f localization and vice versa.

### Band structures

The band structures per spin are calculated using the considered three functionals and two pressures. The results are presented in Table 3 and Fig. 4. The 4f states are also characterized in Fig. 4; the larger thickness of the bands is corresponded to the more contribution of the 4f states. It would be also noted that the E_min_ and E_max_ of the bands in Table 3 are measured with respect to the Fermi energy, i.e., the negative (positive) energies are located below (above) the Fermi level. For convenience, we below start the band structure discussion by considering the results obtained from the most considered localized functional, i.e., B3PW91 with *α* = 0.2 (in this work). Then, we report on the results obtained by the intermediately localized functional, i.e., B3PW91 with *α* = 0.1, and finally concentrate on the least localized functional, i.e., LDA. Thus, the degree of 4f localization is gradually reduced step by step through the discussion presented in this section.

As the first step for describing the band structures using the B3PW91 with *α* = 0.2 at P = 0 and P = 14 GPa, we concentrate only on Fig. 4(a,b) as well as first to third rows of Table 3. The results show that three bands cross the Fermi level at zero pressure which are labeled as *γ*_1_, *γ*_2_ and *γ*_3_. The minimum energy of these bands are negative (E_min_ < 0) while their maximum energies are positive (E_max_ > 0). Their occupancy numbers are also smaller than unity. Applying the pressure causes the bandwidths, occupancy numbers and the shape of the bands to be changed. For example, pressure increases the bandwidth and occupancy number of *γ*_1_. Furthermore, pressure causes the E_max_ of *γ*_1_ and *γ*_2_ bands to decrease which means that pressure pushes these bands downward below the Fermi level. The E_min_ and E_max_ of *γ*_3_ band are also increased by pressure, which means that pressure pushes the *γ*_3_ upward above the Fermi level. But, the pressure cannot cause these bands to be located completely below or above the Fermi level. Thus *γ*_1_, *γ*_2_ and *γ*_3_ cross the Fermi level again at P = 14 GPa. In addition, pressure increases the 4f states around the Fermi level. The thickness of the bands around the Fermi level is larger at P = 14 GPa than P = 0. However, the 4f states at the Fermi level are negligible at the two considered pressures and they are mostly dispersed away from the Fermi level.

We now go to the second step and describe the results with the B3PW91 functional with *α* = 0.1 at P = 0 and P = 14 GPa. Therefore, we concentrate only on Fig. 4(c,d) besides fourth to sixth rows of Table 3. The results show that at zero pressure the E_max_ of *γ*_1_, *γ*_2_ and *γ*_3_ are positive while their E_min_ are negative. Their occupancy numbers are also smaller than unity. These results imply that these three bands cross the Fermi level at zero pressure. This is the same as the situation of the first step. The results reveal an interesting event at P = 14 GPa in this step. The E_max_ of the *γ*_1_ band is negative and its occupancy number is unity at P = 14 GPa. This means that pressure causes the *γ*_1_ to be located below the Fermi level, which is quite different from that of the first step. Pressure also changes the E_min_ and E_max_ of *γ*_2_ and *γ*_3_, but it cannot prevent these bands to cross the Fermi level. Therefore, two bands *γ*_2_ and *γ*_3_ cross the Fermi level at P = 14 GPa compared to the P = 0 situation where three bands (*γ*_1_, *γ*_2_ and *γ*_3_) do this. A manifold of the 4f bands is displayed in Fig. 4(c) which is dispersed near the Fermi level up to 1.5 eV. The same result can be seen at P = 14 GPa. A comparison of Fig. 4(c,d) reveals that the thickness of the bands at Fermi level at P = 14 GPa is larger than those at zero pressure. This implies that the pressure increases the 4f states at the Fermi level.

Let us now consider the last step to describe the LDA results. Therefore, we concentrate on Fig. 4(e,f) together with the seventh to ninth rows of Table 3. The results show that the E_max_ of *γ*_1 _is negative and its occupancy number is unity at zero pressure. This implies that the *γ*_1_ is located below the Fermi level and does not cross it. However, the E_max_s of *γ*_2_ and *γ*_*3*_ are positive and their E_min_s are negative. The occupancy numbers of *γ*_2_ and *γ*_3_ are also smaller than unity. This implies that *γ*_2_ and *γ*_3_ cross the Fermi level. The same results are also obtained at P = 14 GPa. This means that pressure cannot change the number of the bands which cross the Fermi level using the LDA functional. However, pressure changes the position, bandwidths, occupancy numbers and the shape of the bands. For example, pressure increases the bandwidths of the *γ*_1_, *γ*_2_ and *γ*_3_ bands. The results also show a manifold of the 4f bands which straddles the Fermi level at zero pressure. This manifold is dispersed up to 1 eV above the Fermi level. The same result is also seen at P = 14 GPa. This implies that pressure cannot change significantly the position and dispersion of this manifold using the LDA.

In essence, we have shown that the effects of pressure on the band structure can strongly depend on the used functional. This implies that the effects of pressure can be influenced by the degree of 4f localization. Every functional has its own specified degree of 4f localization which will be assumed for and applied on the case under the theoretical study. For example, comparison of Fig. 4(a,c) with (e) shows that the reduction of 4f localization causes the shape of the bands to change and the 4f states to pile up near the Fermi level. The reduction of 4f localization also decreases the bandwidths of *γ*_1_, *γ*_2_ and *γ*_3_ at zero pressure. We conclude the section by the fact that it is crucial to first specify the pressure, and then for each specified pressure use a suitable functional with an appropriate degree of 4f-localization.

### Fermi surfaces

Electronic properties of compounds can be more clearly analyzed by shedding light into their Fermi surface (FS) topologies which contain important experimental information. Several dHvA experiments^40^,^41^,^44^,^45^,^46^ show that the FS of CeIn_3_ compound is changed as pressure increases from P < P_c_ to P ≥ P_c_, which can be explained by changing the character of 4f electrons from localized for P < P_c_ to itinerant for P ≥ P_c_. A variety of approaches have been proposed to describe the FS topology of cerium based compounds. The FS measurements can be well described by the DFT + LDA/GGA band structure calculations in some compounds for which their f-electron shells are completely either filled or empty so that contributions of their f-electrons to the corresponding FSs are negligible. Even if the f-electron shells are neither completely filled nor completely empty, they can be still well described by the DFT + LDA/GGA, provided that the f-electrons of the compounds under question are itinerant and behave as band-like electrons. It was observed that the Ce based compounds with f-itinerant electrons had larger FS than those with f-localized electrons^47^,^48^. These observations revealed that the f-electrons and pressure would play key roles in the FS formation. These have motivated us to investigate the effects of the 4f localization and pressure on the FS of CeIn_3_. Then, we have calculated the FS using the B3PW91 with *α* = 0.2, B3PW91 with *α* = 0.1 and LDA functionals at zero pressure and P = 14 GPa. The FS of a compound includes some branches which are produced by the partially filled bands. The branches of CeIn_3_ Fermi surface are calculated using the three considered functionals for the two pressures and displayed as S_1_, S_2_ and S_3_ in Fig. 5. The S_*j*_ branch is produced by the *γ*_*j*_ band provided that the *γ*_*j*_ band crosses the Fermi level, as expected from the definition of FS. We have also tabulated contributions of the partial DOSs and interstitial (Int) DOS to the DOS(*E*_F_) in Table 4 using these functionals and pressures.

There are three FS branches with the B3PW91 with *α* = 0.2 at P = 0, as depicted in Fig. 5 (a_1*j*_) for *j* = 1 to 3. These three branches also exist at P = 14 GPa using this functional, see Fig. 5 (a_2*j*_) for *j* = 1 to 3. The shapes of these branches at P = 14 GPa are approximately the same as those at P = 0, but the sizes of these branches are changed by pressure. For example, a comparison of Fig. 5(a_11_) and (a_21_) shows that pressure only reduces the sizes of the small packets of S_1_ branch (located in the corners of the first Brillouin zone) and does not drastically change their shapes. Note that the packets shapes remain unchanged, as pressure does not also remarkably change the shapes of the *γ*_1_, *γ*_2_ and *γ*_3_ bands at the Fermi level and the points where these bands cross the Fermi level using this functional, see Fig. 4(a,b). The different sizes of the branches originate from the character change of the bands by pressure. As shown in Table. 3, pressure increases the occupation numbers of *γ*_1_ and *γ*_3_ bands but decreases the occupation number of *γ*_2_ band using the B3PW91 functional with *α* = 0.2. Moreover, the results in Table 4 show that pressure increases the DOS^*f*^(*E*_F_) while it decreases the DOS^*p*^(*E*_F_), DOS^*d*^(*E*_F_) and DOS^*Int*^(*E*_F_) using this functional. The DOS^*s*^(*E*_F_) is also increased by pressure which is negligible with respect to the DOS^*f*^(*E*_F_).

There are also three S_1_,S_2_ and S_3_ branches at zero pressure using the B3PW91 with *α* = 0.1, as shown in Fig. 5 (b_1*j*_) for *j* = 1 to 3. A comparison of Fig. 4(a,c) reveals that the shapes of the *γ*_1_ and *γ*_3_ bands at the Fermi level are not significantly changed by decreasing the *α* parameter at P = 0. This causes the shapes of the S_1_ and S_3_ branches to remain approximately unchanged at P = 0 by reducing *α* parameter from 0.2 to 0.1, compare Fig. 5(a_11_) with (b_11_) and Fig. 5(a_13_) with (b_13_). But, the shape of the *γ*_2_ band is changed at the Fermi level by decreasing the *α* parameter at P = 0. For example, the *γ*_2_ band is touching the Fermi level between *W* and *L* points in Fig. 4(c) while it is not in Fig. 4(a). This causes the shape of the S_2_ branch using the B3PW91 with *α* = 0.2 to be different from that of B3PW91 with *α* = 0.1, compare Fig. 5(a_12_) with (b_12_). This change is due to the degree change of the 4f localization. The results in Table 4 show that the DOS^*f*^(*E*_F_) is significantly increased by decreasing the *α* parameter using the B3PW91 functional while change in the magnitude of the other partial DOSs is negligible at the Fermi level. This causes the character of the bands at the Fermi level and thereby size of the branches to be changed by changing the *α* parameter. These results are in agreement with previous experimental and theoretical measurements^49^ of CeIn_3_ FS at AFM phase. In contrast to the case of P = 0, there are only two S_2_ and S_3_ branches for the case of P = 14 GPa using the B3PW91 with *α* = 0.1, i.e., S_1_ branch is not reproduced at P = 14 GPa after reducing *α*, see Fig. 5 (b_2*j*_) *j* = 1 to 3. Pressure causes the *γ*_1_ band to be located below the Fermi level at P = 14 GPa using the B3PW91 with *α* = 0.1, as discussed in band structures section. This is why the S_1_ branch is disappeared at P = 14 GPa when reducing *α*, see Fig. 5(b_21_). A comparison of Fig. 5(b_12_,b_22_) elucidates that pressure changes the S_2_ branch. This change originates from the effects of pressure on the shape of the *γ*_2_ band at the Fermi level. The *γ*_2_ band is tangent to the Fermi level between *W* and *L* points in Fig. 4(c) while it is not in Fig. 4(d). Pressure also fills the holes of the S_3_ branch at P = 0; the existed empty spaces in Fig. 5(b_13_) are filled in Fig. 5(b_23_) by increasing pressure. The results in Table 4 show that the DOS^*f*^(*E*_F_) is remarkably increased at P = 14 GPa while the DOS^*s*^(*E*_F_), DOS^*d*^(*E*_F_) and DOS^*Int*^(*E*_F_) are decreased with respect to those obtained at P = 0 using the B3PW91 with *α* = 0.1.

Using the LDA functional, as illustrated in Fig. 5 (c_*ij*_) for *i* = 1, 2 and *j* = 1 to 3, there are only two S_2_ and S_3_ branches at P = 0 or similarly at P = 14 GPa, i.e., the S_1_ branch is absent. The *γ*_1_ band is located below the Fermi level within this functional and as a result the S_1_ branch is absent in the FS. The results as presented in Table 4 show that the magnitude of DOS^*f*^(*E*_F_) is much larger than those of the other states. In addition, Fig. 4(e,f) show that the 4f states migrate toward the Fermi level using this functional. These figures also show that the 4f states disperse more uniformly at the Fermi level using the LDA compared to the other functionals. These results indicate that contributions of the 4f states in the two *γ*_1_ and *γ*_2_ bands are high and approximately equal. The shapes of these bands using the LDA are very different from those of the other functionals. Thus, the cross-sectional areas of the FS are filled by LDA much more than the other functionals.

We conclude this section by the following points. There are only two branches for the CeIn_3_ FS with LDA at both P = 0 and P = 14 GPa (in agreement with the experimental results^44^ in the PM phase), while there are three branches in FS obtained by B3PW91 with *α* = 0.2 at both P = 0 and P = 14 GPa as well as the FS obtained by B3PW91 with *α* = 0.1 at P = 0. Although the FSs obtained by B3PW91 with *α* = 0.1 at P = 14 GPa appears to be also in agreement with the experiment by giving only two branches, we notice that 14 GPa is much less than the predicted P_s_ by B3PW91 with *α* = 0.1; B3PW91 failed (even much worse than GGA) to predict the experimental P_c_ (see magnetic properties section). This means that P = 14 GPa is greater than P_s_ using the LDA but less than P_s_ using the B3PW91. This implies that for P = 14 GPa the system lies in the PM phase using the LDA, but it still lies in the AFM phase using the B3PW91. These results reveal that the FSs using those functionals that treat f-electrons as itinerant electrons such as LDA are in better agreement with experiment for P > P_c_ than those functionals that treat f-electrons as localized electrons such as B3PW91. Conversely, FSs using those functionals that treat f-electrons as localized electrons such as B3PW91 are in better agreement with experiment for P~0 than those functionals that treat f-electrons as itinerant electrons such as LDA.

### Electric field gradient

In this section we take the main point of this work into account and show that the electric field gradient (EFG) as an extremely sensitive quantity can be calculated as a function of pressure and show how the EFG can be affected before and after P_c_. The electric field gradient (EFG) is a tensor of rank 2 which has only two independent components in the principal axes system. These components are the main component of EFG, *V*_*zz*_, and the asymmetry parameter, 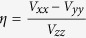. For the case under study, *η* is zero. Thus, we only focus on *V*_*zz*_. The EFG contribution of the electrons inside (at the surface and outside) of the muffin-tin sphere is called the valence (lattice) EFG which is denoted by 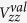 (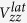). The EFG quantity is extremely sensitive to the anisotropic charge distributions of the core electrons^50^ as well as to the aspherical electron density distribution of valance electrons^51^, and as a result to the valance electronic structure^30^. The EFG, thereby, can serve as a powerful gauge for measuring such a degree of localization^30^.

**Figure 6:**
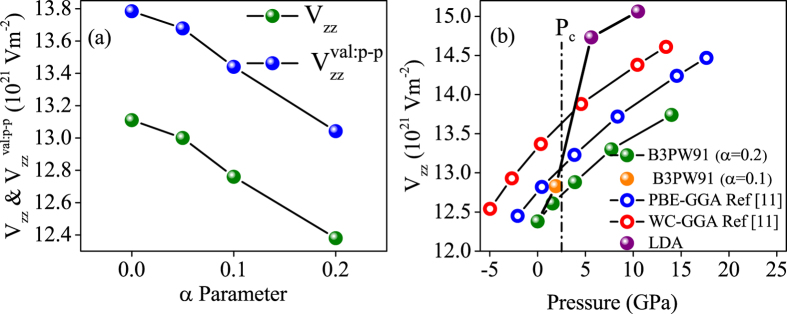
(a) Calculated V_*zz*_ and p-p contribution of the valence V_*zz*_ 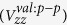 at the In site versus *α* parameter using the B3PW91 functional using equilibrium lattice constant *a* = 4.68 Å which is calculated by the B3PW91 functional with *α* = 0.2 and (b) V_*zz*_ at the In site versus pressure using several functionals.

Here, we calculate the EFGs of CeIn_3_ compound using the B3PW91 with *α* = 0.2, B3PW91 with *α* = 0.1 and LDA functionals. These calculations are performed by taking the equilibrium lattice constant *a* = 4.68 Å into account which is predicted by B3PW91 with *α* = 0.2. The calculated *V*_*zz*_, and their components 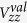 and 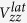 are presented in Table 5 along with the other theoretical and experimental results for comparison. The results show that high 4f localized functionals such as LDA + U and hybrid functionals are more successful in EFG prediction at zero pressure than low 4f localized functionals such as LDA or GGA. This result confirms the high localization degree of CeIn_3_ 4f electrons at zero pressure. Among the high localized functionals, B3PW91 with *α* = 0.2 is in better agreement with experiment than previous theoretical calculations even GGA + U + SO calculations. The success of this functional can be explained by the relation between EFG and total DOS(E_F_). The previous calculations have revealed that EFG ∝ DOS(E_F_)^30^. The total DOS(E_F_) calculated by B3PW91 (*α* = 0.2) is 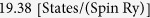 which is smaller than that of other functionals^11^,^3^0 even GGA + U + SO functional^30^. Therefore, EFG using this functional is also smaller and thereby closer to the experiment than those obtained using other functionals. A very small EFGs at Ce site are also reported in Table 5 which is produced by the SOC interactions. Based on the relation EFG ∝ DOS(E_F_) we also anticipate that EFG must increase as the 4f localization decreases, as the DOS(E_F_) is increased by decreasing the 4f localization (see Fig. 3). Our calculations confirm this anticipation. The results reveal that EFG at In site increases as *α* parameter reduces from 0.2 to 0.1 using the B3PW91 functional. Furthermore, the LDA functional with lowest degree of 4f localization yields maximum value of EFG among all considered functionals. Moreover, the previous calculations show that EFG at In site using the PBE-GGA is larger than that of PBE-GGA + U. These results imply that the reduction of 4f localization causes the EFG at In site to increase. To clarify this point, we calculate the EFG at In site with respect to the *α* parameter using the B3PW91 functional, see Fig. 6(a). This figure shows that the EFG increases as the *α* parameter decreases. This figure also shows that the EFG is dominated by the p-electrons. The anisotropy functions of Δ*p*(E_F_) and Δ*d*(E_F_) are also calculated using the three mentioned functionals at P = 0 and P = 14 GPa. The results are tabulated in Table 6. As evidently shown in this table, the magnitude of Δ*p*(E_F_) is one order of magnitude greater than that of Δ*d*(E_F_) at In site. This result is consistent with the previous calculations^30^.

Finally, we have calculated the EFG at In site using the B3PW91 with *α* = 0.2 as a function of pressure, as shown in Fig. 6(b). The results show that EFG increases versus pressure in a bowing shape. This bowing behavior of EFG was also previously seen using the PBE-GGA and WC-GGA^11^. However, as discussed in the previous sections, pressure can change the degree of localization. Hence, it is not appropriate to plot a sensitive quantity such as EFG versus pressure without paying attention to the changes made (by pressure) in the degree of localization. To this end, we have also plotted the EFG curve versus pressure by selecting more appropriate functionals point by point as pressure changes. Thus, EFG is calculated using the B3PW91 with *α* = 0.2 at zero pressure, and using the B3PW91 with *α* = 0.1 at P = 2 GPa, as well as using the LDA at P = 5 and 10 GPa. As it can be clearly seen from Fig. 6(b), the behavior of the EFG curve obtained using a variety of functionals is qualitatively and quantitatively different from those obtained using only a single functional. We close this section by summarizing that a specific functional can be suitable only at a special range of pressure for the strongly correlated system.

### Summary and concluding remarks

We have used the CeIn_3_ compound as an appropriate sample to show how the degree of localization can be strongly affected by pressure in a highly correlated system within the density functional theory. To this end, we have systematically calculated the effects of pressure and 4f localization degree on the structural, magnetic, and electronic properties of this compound. The calculations were performed employing a variety of localized and itinerant functionals versus pressure. Our PBE-GGA results in agreement with the previous results reported by the others predict that the total magnetic moment per cerium atom (TOT) is suppressed for this compound at a pressure of about 16 GPa. However, this prediction of PBE-GGA is very far from the experimental value of 2.5 GPa. This inconsistency between theory and experiment can be improved using LDA functional. The LDA functional yields better results for the suppressed pressure in closer agreement with experiment than those of the other considered functionals. The Fermi surface calculations using the LDA functional are also found to be consistent with the experimental measurements. In addition to the aforementioned inconsistency, the PBE-GGA functional also fails to satisfactorily predict the TOT at zero pressure. The PBE-GGA predicts that the TOT of the compound is 0.24 *μ*_*B*_ at zero pressure, which is inconsistent with the experimental value of 0.65 *μ*_*B*_. In this case, the LDA functional not only dose not improve the predicted TOT at zero pressure, but decreases the accuracy compared to that of PBE-GGA. Our results show that high localized B3PW91 functional with *α* = 0.2 can well predict the TOT of the system at zero pressure (0.67 *μ*_*B*_) in better agreement with the experimental value (0.65 *μ*_*B*_). Lattice parameter and bulk modulus of the system are systematically studied using the GGA + U and B3PW91 functionals by varying their corresponding U_eff_ and *α* parameters, respectively. Our GGA + U calculations show that the bulk modulus of the system unreliably fluctuates as U_eff_ parameter increases. This behavior which does not allow to select an appropriate value for the U_eff_ indicates that the DFT + U scheme is rather unreliable. The hybrid B3PW91 approach provides much better agreement with the experimental data than the GGA + U scheme. The lattice parameters (bulk moduli) for the AFM phase are calculated to be 4.69 Å (52.41 GPa), 4.68 Å (61.77 GPa), and 4.68 Å (62.30 GPa) by B3PW91 with *α* = 0.4, 0.3, and 0.2, respectively. The comparison of these results and the corresponding experimental data, 4.69 Å (67.00 GPa), shows that the best lattice parameter is obtained using a larger value of *α* (0.4) than that of usually considered for the AFM phase, see Table 1. Our results show that the GGA + U fails to produce acceptable results by increasing the U_eff_ parameter since not only its predicted bulk modulus unpredictably fluctuates by increasing U_eff_ but also the fluctuations are performed around a value which are far from the experimental bulk modulus. However, it is worth mentioning that the hybrid B3PW91 gives reliable results for the AFM phase of the system using the larger value of *α* = 0.3. The latter result together with the excellent prediction of the lattice parameter by even a larger value of α (0.4) in agreement with the previous study of CuCl_2_ compound^34^ confirms that the percentage of HF would be increased for obtaining more reliable results. Therefore, it is crucial to calibrate the amount of HF exchange for predicting reliable results. By following this strategy after a systematic study we found that bulk modulus of the system in its AFM phase is well predicted by *α* = 0.2 and 0.3. By considering both of the lattice parameter and bulk modulus results and calibrating them to the corresponding experimental data we found that the B3PW91 with *α* = 0.2 can predict the structural properties more consistent with the experimental data. The agreement with the experimental results is acceptable with the standard B3PW91 approach for the FM phase. It is worth mentioning that these consistencies can only partially validate the correctness of the reported results. Furthermore, the Fermi surface calculations using this functional are in agreement with experimental measurements for the AFM phase. In addition, the calculated electric field gradients at In sites by this hybrid functional are found to be very closer to the experimental data at zero pressure compared to the other considered functionals. However, this hybrid functional makes the predicted suppressed pressure worse than that of PBE-GGA compared to the experimental result. Thus, the high localized B3PW91 functional with *α* = 0.2 is suitable functional in predicting the CeIn_3_ properties at zero pressure while the LDA is a suitable functional for high pressures. This means that the character of 4f electrons is changed by applying the pressure in this compound in agreement with the dHvA experiments. Therefore, the degree of 4f localization must be tuned in terms of the pressure. High localization is suitable for low pressures and vice versa; viz., low localization is suitable for high pressures. Based on these results we conclude that the GGA failure originates from the degree of 4f localization which is considered in GGA functional. The GGA is not low localized enough to become suitable for studying the CeIn_3_ at high pressures. The 4f-electrons are more localized using the GGA than those of LDA and thereby GGA predicts the critical pressure much larger than those of experiment and LDA. On the other hand, GGA is not highly localized enough to become suitable for studying the CeIn_3_ at very low pressures around the P = 0. Thus, the TOT is predicted by the PBE-GGA functional at zero pressure to be smaller than those of the experiment and B3PW91 functional with *α* = 0.2. Our electronic structure, including DOS, band structure, and Fermi surface analyses reveal that if a fixed exchange-correlation functional is used for every pressure, then the effects of pressure on the electronic structure will not be well considered for the highly correlated systems. This is in contrast to what happens in nature, because if the degree of 4f-electron localization decreases, the electronic structure can be changed. Our results show that the GGA functional can be considered as an appropriate functional for studying the CeIn_3_ compound at intermediate pressures but not for every pressure regime. In essence, this state-of-the-art DFT study demonstrates that the variation of a physical quantity with respect to pressure can sensitively depend on the used exchange-correlation functional in such a system. This in turn implies that the behavior of a physical quantity as a function of pressure can be valid within DFT only over a specific pressure range. Therefore, a physical quantity-versus-pressure curve cannot always be obtained reliably by only one currently available exchange-correlation functional over an arbitrary range of pressure. Instead, it would be more reliable to first divide the arbitrary pressure interval into some specific pressure intervals. Then, the degree of localization is determined for each pressure interval. Eventually, based on the latter determination the best exchange-correlation functional is selected and/or tuned for every pressure interval. Otherwise, consistency between experimental and theoretical DFT results using a specifically fixed exchange-correlation functional cannot be guaranteed for any pressure interval so that even one may expect to encounter an extraordinary large discrepancy between theory and experiment. By this strategy, we have explained why there are two dramatic discrepancies between previous reliable experimental and accurate *ab initio* DFT results in CeIn_3_ and discussed how these discrepancies can be resolved.

## Additional Information

How to cite this article: Yazdani-Kachoei, M. *et al*. Pressure dependency of localization degree in heavy fermion CeIn_3_: A density functional theory analysis. *Sci. Rep.* 6, 31734; doi: 10.1038/srep31734 (2016).

## Figures and Tables

**Table 1:** Calculated lattice parameter (*a*) and bulk modulus (B_0_) of CeIn_3_ in the ferromagnetic (FM) and antiferromagnetic (AFM) phases by a variety of exchange-correlation functionals (XCFs) together with the previous experimental and theoretical data.

XCF	AFM		FM	
	*a* (Å)	B_0_ (GPa)	*a* (Å)	B_0_ (GPa)
LDA	4.55	71.00	4.55	71.9
PBE-GGA	4.69	56.20	4.69	55.8
PBE-GGA^a^	4.70	55.20		
WC-GGA^a^	4.62	66.46		
GGA + U (U_eff_ = 0.5 eV)	4.70	51.27	4.70	51.63
GGA + U (U_eff_ = 1 eV)	4.71	51.89	4.71	52.04
GGA + U (U_eff_ = 1.5 eV)	4.72	49.40	4.72	50.32
GGA + U (U_eff_ = 2 eV)	4.72	51.83	4.72	52.14
GGA + U (U_eff_ = 2.5 eV)	4.73	51.03	4.73	52.98
GGA + U (U_eff_ = 3 eV)	4.73	52.40	4.73	52.09
GGA + U (U_eff_ = 3.5 eV)	4.74	51.47	4.73	51.85
GGA + U (U_eff_ = 4 eV)	4.74	50.68	4.74	51.30
GGA + U (U_eff_ = 4.5 eV)	4.74	46.24	4.74	51.50
GGA + U (U_eff_ = 5 eV)	4.75	52.40	4.74	51.81
GGA + U (U_eff_ = 5.5 eV)	4.75	55.22	4.74	52.33
LDA + U (U_eff_ = 5.5 eV)	4.60	69.31	4.61	65.18
B3LYP (*α* = 0.2)	4.78	31.10	4.77	47.00
B3PW91 (*α* = 0.4)	4.69	52.41	4.66	59.30
B3PW91 (*α* = 0.35)	4.68	53.18	4.68	57.18
B3PW91 (*α* = 0.3)	4.68	61.77	4.64	58.94
B3PW91 (*α* = 0.25)	4.68	56.36	4.69	56.33
B3PW91 (*α* = 0.2)	4.68	62.30	4.68	69.00
B3PW91 (*α* = 0.15)	4.67	56.47	4.68	57.018
B3PW91 (*α* = 0.1)	4.67	61.30	4.67	58.30
B3PW91 (*α* = 0.05)	4.64	57.01	4.65	55.16
B3PW91 (*α* = 0.0)	4.64	60.64	4.64	62.27
EECE	4.74	36.94	4.64	65.41
Exp.	4.69b,^c^	67.00^d^		

^a^Reference [11].

^b^Reference [32].

^c^Reference [33].

^d^Reference [52].

**Table 2:** Calculated total magnetic moment (TOT) per Ce atom of CeIn_3 _compound in the *μ*_*B*_ unit for several pressures within several exchange-correlation functionals (XCFs).

GGA96^a^			WC-GGA^b^				PBE-GGA^b^			PBE-GGA			LDA			B3LYP			EECE			
P(GPa)	TOT		P(GPa)		TOT		P(GPa)	TOT		P(GPa)	TOT		P(GPa)	TOT		P(GPa)	TOT		P(GPa)	TOT		
0.0	0.18		0.0		0.11		0.0	0.17		−4.1	0.28		−6.4	0.07		0.09	0.71		−0.9	0.46		
			9.0		0.00		16.0	0.00		−3.3	0.09		−5.1	0.08		1.2	0.72		−0.5	0.45		
										0.0	0.23		3.7	0.20		2.7	0.72		1.7	0.43		
										7.4	0.17		−1.1	0.07		4.7	0.71		6.7	0.40		
										13.6	0.06		0.0	0.00		7.4	0.70		15.8	0.35		
										16.8	0.00		3.5	0.00		12.2	0.69					
PBE-GGA + U																						
U_eff_ = 0.5 eV		U_eff_ = 1 eV		U_eff_ = 2.5 eV			U_eff_ = 1.5 eV		U_eff_ = 2 eV		U_eff_ = 3 eV		U_eff_ = 3.5 eV		U_eff_ = 4.5 eV		U_eff_ = 4.5 eV		U_eff_ = 5 eV		U_eff_ = 5.5 eV	
P(GPa)	TOT	P(GPa)	TOT	P(GPa)		TOT	P(GPa)	TOT	P(GPa)	TOT	P(GPa)	TOT	P(GPa)	TOT	P(GPa)	TOT	P(GPa)	TOT	P(GPa)	TOT	P(GPa)	TOT
−4.9	0.54	−4.9	0.72	−4.9		0.79	−2.9	0.82	−4.7	0.86	−3.2	0.84	−4.6	0.89	−2.8	0.88	−2.4	0.89	−2.6	0.89	−5.2	0.92
−3.8	0.20	−3.6	0.68	−3.3		0.76	−3.0	0.31	−3.2	0.84	−1.1	0.81	−1.0	0.84	−0.9	0.86	−0.9	0.72	−1.0	0.88	−3.6	0.90
−2.1	0.21	−1.8	0.62	−1.4		0.72	−2.1	0.75	−1.2	0.81	1.5	0.78	1.6	0.82	5.0	0.79	0.9	0.85	2.2	0.67	−0.6	0.89
0.1	0.17	0.8	0.57	1.0		0.65	−0.2	0.71	1.3	0.77	4.8	0.74	4.9	0.77	9.6	0.76	5.0	0.81	5.9	0.18	5.0	0.84
3.2	0.21	3.8	0.53	3.9		0.60	4.1	0.66	4.5	0.72	9.0	0.70	9.2	0.74	15.9	0.73	10.3	0.77	9.9	0.31	13.7	0.80
7.3	0.19	8.0	0.46	8.7		0.54	11.2	0.62	8.8	0.69							16.1	0.76	16.2	0.25		
13.4	0.17	13.7	0.34				15.5	0.51														
B3PW91																						
*α* = 0		*α* = 0.05		*α* = 0.10			*α* = 0.15		*α* = 0.20		*α* = 0.25		*α* = 0.30		*α* = 0.35		*α* = 0.4					
P(GPa)	TOT	P(GPa)	TOT	P(GPa)		TOT	P(GPa)	TOT	P(GPa)	TOT	P(GPa)	TOT	P(GPa)	TOT	P(GPa)	TOT	P(GPa)	TOT				
−5.7	0.21	−5.5	−0.02	−4.0		0.60	−4.8	0.59	−3.5	0.67	−4.5	0.32	−2.5	0.35	−3.8	0.77	−3.8	0.57				
−4.1	0.20	−3.8	0.00	3.2		0.55	−2.9	0.59	0.0	0.67	−2.7	0.32	−0.1	0.34	−0.5	0.46	−3.8	0.57				
−1.9	0.19	−1.6	0.02	6.4		0.51	−0.5	0.57	3.9	0.65	−0.3	0.32	2.4	0.35	0.0	0.46	−2.2	0.39				
5.2	0.14	1.3	0.03	11.1		0.45	1.9	0.55	7.7	0.63	2.9	0.32	2.9	0.35	3.3	0.45	0.1	0.39				
1.1	0.17	5.1	0.06	23.4		0.29	6.6	0.51	33.4	0.57	7.2	0.32	5.0	0.57			7.5	0.39				
EXP^c^																						
						P(GPa)											TOT					
						0.0											0.65					

^a^Reference [12].

^b^Reference [11].

^c^Reference [13].

**Table 3:** Maximum energy (E_min_) and minimum energy (E_max_), bandwidth and occupancy number (Occup) of three *γ*_1_, *γ*_2_ and *γ*_3_ bands using the B3PW91 with *α* = 0.2 and *α* = 0.1 as well as LDA at P = 0 and P = 14 GPa.

XCF	Label	E_min_(eV)		E_max_(eV)		Bandwidth		Occup	
		P = 0	P = 14 GPa	P = 0	P = 14 GPa	P = 0	P = 14 GPa	P = 0	P = 14 GPa
B3PW91 ( = 0.2)	*γ*_1_	−1.078	−1.220	0.116	0.043	1.194	1.263	0.997	0.999
	*γ*_*2*_	−1.046	−1.204	0.221	0.196	1.267	1.400	0.905	0.868
	*γ*_*3*_	−1.020	−0.781	0.518	0.676	1.538	1.457	0.098	0.133
B3PW91 ( = 0.1)	*γ*_1_	−1.063	−1.230	0.051	−0.027	1.114	1.203	0.997	1.000
	*γ*_2_	−1.031	−1.225	0.152	0.090	1.183	1.315	0.858	0.819
	*γ*_3_	−0.263	−0.161	0.319	0.341	0.582	0.502	0.145	0.181
LDA	*γ*_1_	−1.064	−1.237	−0.060	−0.089	1.004	1.148	1.000	1.000
	*γ*_2_	−1.030	−1.219	0.096	0.205	1.126	1.424	0.820	0.783
	*γ*_3_	−0.067	−0.106	0.119	0.217	0.186	0.323	0.180	0.218

E_min_ and E_max_ are measured with respect to the Fermi level so that positive energies are located above the Fermi level while negative energies are located below the Fermi level.

**Table 4:** The magnitude of s, p, d, f partial DOSs and DOS in the interstitial region (Int) at the Fermi level within several exchange-correlation functionals and two pressures.

Shell	P (GPa)	DOS(E_F_)[States/(eVSpin)]		
		B3PW91 (*α* = 0.2)	B3PW91 (*α* = 0.1)	LDA
s	0	0.15	0.14	0.09
	14	0.16	0.10	0.13
p	0	0.31	0.33	0.35
	14	0.28	0.34	0.34
d	0	0.40	0.43	0.36
	14	0.35	0.35	0.41
f	0	0.52	1.94	7.20
	14	0.61	4.02	3.06
Int	0	0.53	0.63	0.81
	14	0.21	0.40	0.37

The partial DOSs include the contributions of all atoms.

**Table 5:** The main component of EFG, V_*zz*_, besides its valence, 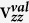, and lattice, 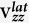, components in 10^21^*Vm*^−2^ unit at Ce and In sites using several exchange-correlation functionals (XCFs).

XCF	V_*zz*_		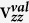		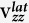	
	In	Ce	In	Ce	In	Ce
PBE-GGA^a^	12.446	0.055	12.496	0.049	−0.05	0.006
WC-GGA^a^	13.152	0.049	13.208	0.047	−0.056	0.002
PBE-GGA^b^	12.857	0.029	12.961	0.040	−0.004	−0.011
PBE-GGA + U^b^	12.431	−2.863	12.442	−2.889	−0.011	0.026
B3PW91(*α* = 0.2)	12.380	−3.114	12.393	−3.110	−0.013	−0.004
B3PW91 (*α* = 0.1)	12.760	−2.362	−2.359	12.772	−0.003	−0.012
LDA	13.310	0.066	13.320	0.066	−0.010	0.000
EXP^c^	11.6					

^a^Reference [11].

^b^Reference [30].

^c^Reference [53].

Calculations in the present work are done using the equilibrium lattice constant *a* = 4.68 Å as evaluated by B3PW91with *α* = 0.2.

**Table 6:** Valence p and d anisotropy functions, Δ*p* and Δ*d*, evaluated at Ce and In sites using the B3PW91 with *α* = 0.2 and *α* = 0.1 as well as LDA at P = 0 and P = 14 GPa.

Method	P = 0				P = 14 GPa			
	Δ*p*		Δ*d*		Δ*p*		Δ*d*	
	In	Ce	In	Ce	In	Ce	In	Ce
B3PW91 (*α* = 0.2)	−0.0182	0.0042	0.00270	0.017	−0.0206	0.0663	0.00195	0.01512
B3PW91 (*α* = 0.1)	−0.0186	0.0031	0.0028	0.0124	−0.0187	0.0350	0.0031	0.0083
LDA	−0.0189	0.0009	0.0023	−0.0008	−0.0195	0.0003	0.0027	−0.0004
